# Longitudinal amyloid-beta measurements and blood–brain barrier dysfunction in cerebral amyloid angiopathy as part of the Mechanisms Of DEMentia project: protocol of the MODEM-LAMBDA-C study

**DOI:** 10.3389/fneur.2026.1949765

**Published:** 2026-10-28

**Authors:** Arno Stellingwerf, Floris H. B. M. Schreuder, H. Bea Kuiperij, Anja G. Van Der Kolk, Walter H. Backes, Jurgen A. H. R. Claassen, Marcel M. Verbeek

**Affiliations:** 1 Department of Neurology, Donders Institute for Brain, Cognition and Behaviour, Radboud University Medical Centre, Nijmegen, Netherlands; 2 Radboud Alzheimer Centre, Radboud University Medical Centre, Nijmegen, Netherlands; 3 Department of Medical Imaging, Radboudumc, Nijmegen, Netherlands; 4 Department of Radiology & Nuclear Medicine, Maastricht University Medical Center+, Maastricht, Netherlands; 5 Mental Health and Neuroscience (MHeNs) Research Institute, Maastricht University, Maastricht, Netherlands; 6 Cardiovascular Research Institute Maastricht (CARIM), Maastricht University, Maastricht, Netherlands; 7 Department of Geriatric Medicine, Radboud University Medical Centre, Nijmegen, Netherlands; 8 Department of Cardiovascular Sciences, University of Leicester, Leicester, United Kingdom; 9 Department of Human Genetics, Radboud University Medical Centre, Nijmegen, Netherlands

**Keywords:** amyloid-beta, blood–brain barrier dysfunction, cerebral amyloid angiopathy, cerebrospinal fluid, DCE-MRI, longitudinal

## Abstract

**Background:**

Cerebral amyloid angiopathy (CAA) is characterized by the deposition of amyloid-beta (Aβ) peptides within the cerebral vasculature and is a major cause of lobar intracerebral haemorrhage, cognitive impairment, and dementia. Vascular Aβ aggregates are enriched in shorter peptides (Aβ38 and Aβ40) whereas neuritic plaques, as observed in Alzheimer’s disease pathology, predominantly contain longer Aβ peptides (Aβ42 and Aβ43). These pathological signatures are reflected by reduced cerebrospinal fluid (CSF) Aβ levels. However, the longitudinal dynamics of CSF Aβ peptides in CAA and their relationship with blood–brain barrier (BBB) dysfunction remain poorly understood.

**Methods:**

The MODEM-LAMBDA-C study is a longitudinal observational study including participants with CAA and age- and sex-matched control participants. At baseline, participants undergo clinical assessment, cognitive testing, blood pressure measurement, dynamic contrast-enhanced magnetic resonance imaging (DCE-MRI) of the brain, blood sampling, and lumbar puncture to obtain CSF. At 2 years of follow-up, all assessments except MRI are repeated. Longitudinal changes in CSF Aβ peptide concentrations will be examined and related to cognitive performance, baseline MRI markers of cerebral small vessel disease and DCE-MRI-derived measures of BBB leakage. Additionally, CSF biomarkers of BBB dysfunction will be quantified and correlated with DCE-MRI markers of BBB dysfunction.

**Conclusion:**

By integrating biochemical and imaging markers of Aβ pathology and BBB dysfunction, the MODEM-LAMBDA-C study aims to provide a comprehensive understanding of disease mechanisms underlying CAA, potentially contributing to improved diagnosis and prognostic strategies.

**Clinical trial registration:**

https://onderzoekmetmensen.nl/en, identifier: NL85709.091.23.

## Introduction

Cerebral amyloid angiopathy (CAA) is a major cause of lobar intracerebral haemorrhage (ICH), convexity subarachnoid haemorrhage, cognitive impairment and dementia (1). In CAA, amyloid-beta (Aβ) peptides are deposited predominantly in the arterioles, leptomeningeal vessels, and in capillaries of the cerebral cortex. The exact mechanisms initiating Aβ-aggregation and propagation are currently not fully understood but certain risk-factors, such as APOE variants, high blood-pressure, and brain injury are known (2–4). The pathophysiological mechanism underlying Alzheimer’s disease (AD) is hypothesized to involve a disruption in the balance between Aβ production, resulting from enzymatic cleavage of amyloid precursor protein (APP), and Aβ clearance. APP cleavage may result in Aβ peptides of varying length of which shorter Aβ peptides (e.g., Aβ38, Aβ40) are more soluble than longer Aβ peptides (e.g., Aβ42, Aβ43). After its production, the Aβ peptides are present in the brain interstitial fluid and may be cleared through several mechanisms: (1) drainage along perivascular pathways, including intramural peri-arterial drainage (IPAD) (5, 6), eventually reaching meningeal and cervical lymphatic systems; (2) clearance via the glymphatic system (6, 7); (3) transport across the blood–brain barrier (BBB) mediated by transport proteins such as LRP1 and P-glycoprotein (8); and (4) enzymatic degradation and cellular uptake by microglia and astrocytes (9). Impairment of one or more of these clearance mechanisms may contribute to cerebral Aβ accumulation at distinct locations (6, 10). Shorter Aβ peptides are more associated with vascular deposits, whereas the longer Aβ peptides accumulate in neuritic plaques, as found in Alzheimer’s disease (AD) (11–14). It is important to note that CAA and AD pathology often co-occur since moderate-to-severe CAA is present in almost 50% of the AD patients (15). The aggregation of these different Aβ peptides is reflected by reduced concentrations of these peptides in cerebrospinal fluid (CSF). A meta-analysis of cross-sectional studies showed that CSF levels of Aβ38, Aβ40, and Aβ42 are decreased in CAA compared to controls (16), and it has been shown that Aβ43 is also reduced in CSF of CAA patients (17). The CSF Aβ-profile is also different between CAA and AD since Aβ38, Aβ40 and Aβ42 concentrations are decreased in CAA while Aβ43 levels are similar (17).

Improved understanding of the pathophysiology of these peptides could turn them into established fluid biomarkers indicating the presence of CAA, also in the presence of co-existing AD pathology. Improved knowledge of the longitudinal changes of these CSF Aβ peptides is critical since they can provide insights in the mechanisms of CAA propagation, disease prognosis, and aid with the detection of asymptomatic CAA pathology in AD. This is critical since with the emergence of anti-Aβ therapies in AD, it has been shown that pre-existing CAA is a risk factor for developing clinical complications of this therapy (18). Furthermore, CAA biomarkers could aid in early disease stage diagnosis, risk management, and clinical decision making regarding the use of anti-coagulants (1, 16).

One of the hypothesized causes for Aβ accumulation in CAA is dysfunction of the BBB (19, 20). BBB dysfunction is defined as changes in bidirectional permeability via any of the BBB’s transport routes, emphasizing its natural selectivity that is impaired (21). The gold standard for identifying BBB dysfunction is post-mortem immunohistochemical examination of extravasated serum proteins, such as fibrinogen, thrombin, and albumin, in the perivascular brain parenchyma (20, 22–24).

At present, it is challenging to properly monitor BBB dysfunction in people with CAA during life since fluid biomarkers are not well defined (21, 25–28). There are no established fluid BBB-biomarkers that reflect specifically the barrier (dys)function, e.g., by showing altered permeability or leakiness of the BBB, and/or reflect BBB-molecular defects, e.g., changes in expression of structural BBB-associated proteins reflecting the function or maintenance of the barrier function (21). Currently, the ratio of the albumin concentration of CSF over serum (Qalb) is commonly used as BBB-dysfunction indicator, however this marker lacks specificity (27, 29–32).

As an *in vivo* alternative biomarker for BBB dysfunction, dynamic contrast-enhanced magnetic resonance imaging (DCE-MRI) has been used to determine the leakage of gadolinium-based contrast agents as a proxy for BBB permeability and, indirectly, (dys)function. However, two recent studies, with a total of 39 participants with CAA, have shown conflicting results (33, 34). Furthermore, to date, no studies have been published with correlations between fluid BBB-biomarkers and DCE-MRI in patients with CAA. Therefore, it is not established if CSF Aβ levels associate with BBB dysfunction, as measured with orthogonal methods, and with clinical outcomes, such as cognitive decline and haemorrhagic manifestations in patients with CAA.

The MODEM-LAMBDA-C study aims to compare the longitudinal changes of Aβ38, Aβ40, Aβ42, and Aβ43 concentrations in CSF between patients with CAA and cognitive healthy individuals without CAA. Additionally, we aim to assess associations of these CSF-Aβ profiles with BBB dysfunction, as measured with DCE-MRI and fluid BBB-biomarkers.

## Methods and analysis

### Study design and ethics

This is a single-centre, prospective, longitudinal observational cohort study with 2–4 years follow-up, carried out at the Radboudumc in Nijmegen, the Netherlands. The study will be conducted according to the principles of the Declaration of Helsinki (35) and in accordance with the Medical Research Involving Human Subject Act (WMO) (35). This study was approved by the medical ethics committee (METC) Oost-Nederland (reference NL85709.091.23). No benefits are identified for the participants and due to the absence of an intervention, a treatment effect is not applicable. Written informed consent will be obtained from all participants after detailed explanation of the study procedures.

### Objectives

The primary objective of this study is to compare the longitudinal changes of Aβ38, Aβ40, Aβ42, and Aβ43 concentrations in CSF between patients with CAA and age- and sex-matched control participants.

The secondary objectives of this study are (1) to cross-sectionally describe whether the CSF Aβ peptide concentrations correlate with (novel) fluid BBB-dysfunction biomarkers and establish if these correlations differ between CAA and control participants. The novel fluid biomarkers candidates will be selected based on proven functional relationship with BBB function, the expression specificity, and if the fluid biomarker can be determined in CSF or blood (21); (2) to determine the integrity of the BBB measured with DCE-MRI in CAA and control participants; (3) to study if the DCE-MRI vascular leakage correlates with both Aβ-peptides and fluid BBB-dysfunction biomarkers in CAA and control participants, and; (4) to study whether the variants of the *APOE* gene are associated with measured levels of the Aβ peptides and fluid BBB-dysfunction biomarkers.

### Recruitment, informed consent and participants

This study builds upon two cross-sectional studies conducted priorly at the Radboudumc: the BIONIC and CAFÉ studies (METC files 2017–3,810 and 2017–3,605) (17). In these previous studies, CSF and blood samples were collected from 46 participants with CAA and 29 control subjects. In the MODEM-LAMBDA-C study, we will re-invite these participants for a second visit to allow for longitudinal collection of CSF and blood samples and perform DCE-MRI imaging. We expect 20 participants with CAA and a similar number of control participants to enrol in this longitudinal follow-up project. An interim overview of the current recruitment, as of mid-October 2026, is provided in Supplementary Table S3.

Additionally, we aim to enroll 10 new participants with CAA and 10 healthy controls, to reach a total 30 participants per group. At the Radboudumc neurology outpatient clinic we will recruit patients with a diagnosis of CAA, according to the Boston 2.0 criteria (36). The control participants will be recruited via an online recruitment platform for brain research (hersenonderzoek.nl). Control participants will be age- and sex matched to the CAA patients. In Table 1 we show the timetable of both the BIONIC/CAFÉ study and the current MODEM study.

**Table 1:** Gantt chart of the BIONIC/CAFÉ study and the MODEM-LAMBDA-C study showing the period of recruitment and follow-up visits.

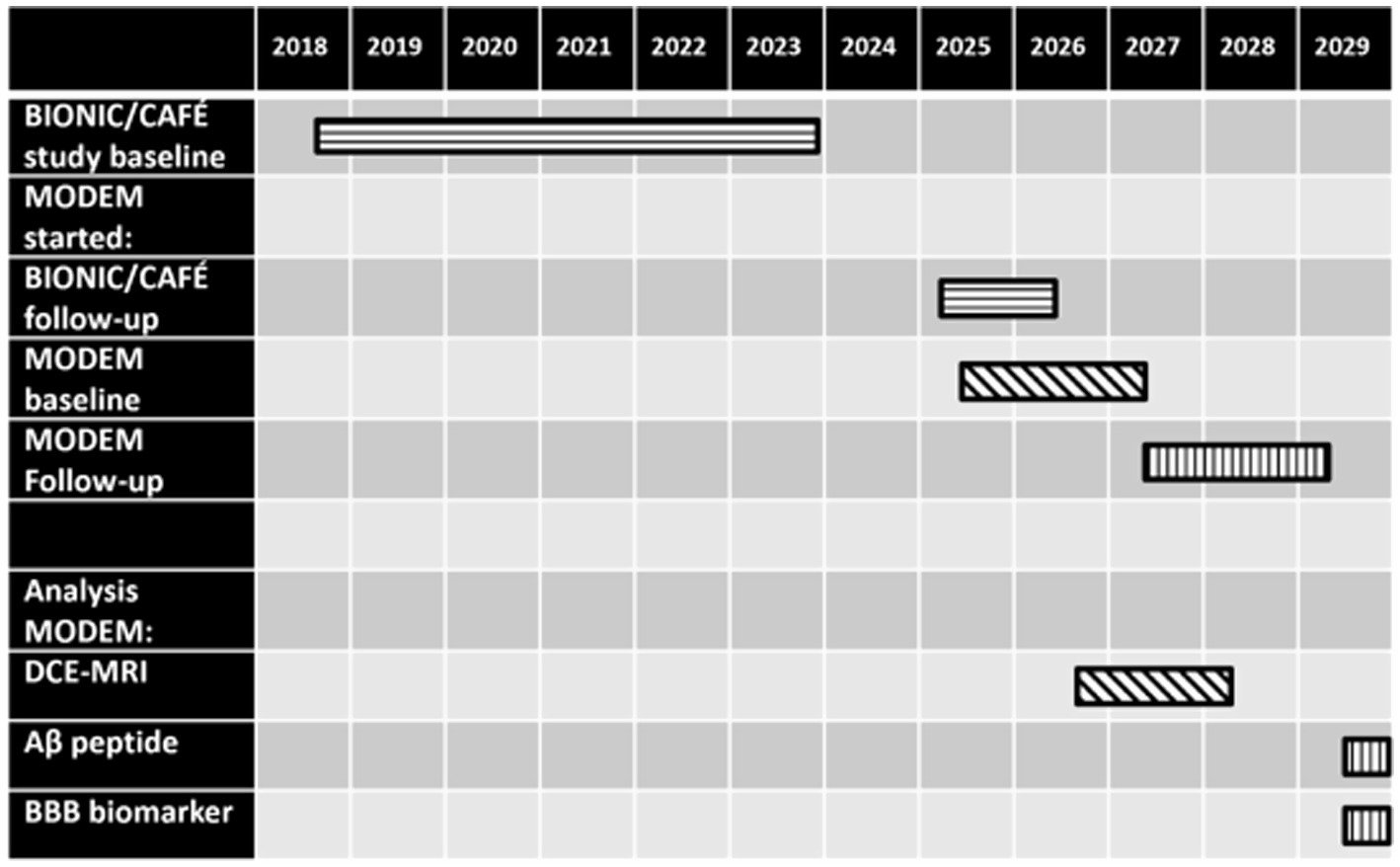

Also, planned analysis of DCE-MRI, CSF CAA biomarkers (the amyloid peptides and Tau proteins), and the BBB-biomarkers are shown. In the time-bars are horizontal lines representing finished milestones, diagonal lines in process projects and vertical lines, not yet started projects.

#### Sample size

This is an explorative study, and as there is no longitudinal data on changes in CSF Aβ concentrations in patients with CAA. For this reason, a formal sample size calculation was not possible to perform reliably. We aim to include a final number of 25 participants in both the CAA and control group. To cope with potential withdrawals and loss-to-follow-up, we increased the required sample size with 20% and we aim to include *n* = 30 per group. Reasons for drop-out may include ICH recurrence in the CAA group [expected rate of 8% per year (37)], cognitive decline in the control group, and non-successful lumbar punctures (estimated at 10%) (17).

#### Eligibility criteria

The eligibility criteria for both the patients with CAA and the control participants are described in Supplementary Tables S1, S2 contains these criteria of the previously conducted BIONIC/CAFÉ study. For control participants, immediately after informed consent a cognitive test is performed to assess eligibility for inclusion, since performing a cognitive test and recording these results is not permitted before informed consent.

### Study procedures

The on-site performed study procedures are identical for both participants with CAA and controls. The procedures are performed by trained research staff as registered in the delegation log. An overview of the study procedures is provided in Table 2. We aimed to harmonize the MODEM-LAMBDA-C study as much as possible with the previously conducted BIONIC/CAFÉ studies by using identical procedures for obtaining baseline characteristics, sample collection and processing, and clinical assessments.

**Table 2:** Overview of the study related actions in chronologic order for each group.

	Participants seen previously in BIONIC/CAFÉ study		Newly recruited participants	
	Control	CAA	Control	CAA
Enrolments and telephonic screening	x	x	x	x
Baseline visit	Study procedures were performed during BIONIC/CAFÉ project		x	x
informed consent			x	x
MoCA			x	x
Symptoms questionnaire			x	x
Blood pressure			x	x
(DCE-)MRI			x	x
Venepuncture			x	x
Lumbar puncture			x	x
Telephone check 1 week after visit			x	x
Second visit > 2 years after baseline visit of BIONIC/CAFÉ or MODEM				
TICS-M			x	
Informed consent	x	x		
MoCA	x	x	x	x
Symptoms questionnaire	x	x	x	x
Blood pressure	x	x	x	x
(DCE-)MRI	x	x		
Venepuncture	x	x	x	x
Lumbar puncture	x	x	x	x
Telephonic check 1 week after visit	x	x	x	x

#### Cognitive assessment

All participants will undergo a standardized assessment of global cognitive function at both visits using the Montreal Cognitive Assessment (MoCA) (38). Approximately 2 months prior to the follow-up visit of newly recruited control participants, we will perform a short modified interview for cognitive status via telephone (TICS-M) (39) to assure no relevant cognitive decline has occurred since the primary visit 2 years earlier. If a control participant scores ≤27 points in the MoCA or ≤34 on the TICS-M test, this person will be excluded from (further) participation.

#### Questionnaires

At baseline and follow-up visits, participants will be interviewed with standardised questionnaires on demographics, the occurrence of relevant neurological symptoms [i.e., ICH, transient focal neurological episodes (TFNE), and cognitive complaints], prior medical history, family history, medication use, and substance abuse. A history of significant head trauma, episodes of migraine, sleeping disorders, intracranial neurosurgery and intracranial radiotherapy will be binary (yes/no) recorded while the option “unknown” is also available if the participant is unsure. General sleep quality and duration will be recorded but not incorporated in any further analysis in this study. Regarding family history, we ask if cardiovascular and/or dementia runs in the family. The questionnaires are available as Supplementary file 2.

#### Blood pressure measurement

Blood pressure (BP) will be measured following the recommendations of the Australian National Heart Foundation (NHF) (40). Patients will rest seated for 5 min, an appropriate cuff size will be selected, and two measurements of BP will be recorded, each arm once and 5 min apart, using an Omron or equivalent validated automated digital BP monitor. Length and weight will also be measured.

#### MRI examination

Participants will undergo a 3T brain MRI examination during the initial visit of the MODEM-LAMBDA-C study (32-channel head coil, MAGNETOM PrismaFit, Siemens Healthineers, Erlangen, Germany). The following sequences will be acquired: 3D T_1_-weighted magnetization-prepared 2 rapid gradient echo (MP2RAGE) for quantification of the tissue T_1_ relaxation times, 3D T_2_-weighted fluid-attenuated inversion recovery (FLAIR) for detection of white matter hyperintensities, diffusion-weighted imaging (DWI), 3D quantitative susceptibility mapping (QSM) for CAA extent and quantification of susceptibility as a proxy of neuroinflammation-associated iron-rich macrophages, and a dynamic contrast-enhanced (DCE) sequence. The DCE sequence, used for assessment of BBB leakage, has been performed at the Radboudumc, and this protocol has been adopted for the current study (41, 42). The sequence consists of five pre-bolus spoiled gradient-recalled-echo (SPGR) scans, then one bolus of Gadovist (gadobutrol, Bayer, Germany) will be administered intravenously (0.1 mL/kg, 3 mL/s) with simultaneous 32 fast SPGR scans, and immediately following 70 acquisitions over a period of 25 min. Acquisition parameters of the used sequences are shown in Supplementary Table S4.

#### MRI analysis

We will assess, as recommended by the Boston 2.0 criteria (36), the presence and extent of cortical superficial siderosis (cSS) with QSM (43), the number and distribution of cerebral microbleeds (CMBs) by QSM (44), and white matter hyperintensities (WMH) with FLAIR according to the Fazekas scale (45). The small vessel disease burden (SVD) summary score, ranging from 0 to 6 points, will be based on these four parameters (46). The leakage rate *K_i_* [min^−1^], the fractional tissue volume of leaking microvessels (*V_i_*) and the fractional blood plasma volume (*V_p_*) will be obtained from the DCE imaging (41) via kinetic (Patlak plot) analysis using the DCE signal time-series of the tissue and superior sagittal sinus (vascular input function) as input variables.

#### Lumbar puncture, blood sampling and (pre-)analytical procedures

During the baseline and follow-up visits, approximately 15 mL of CSF will be collected in polypropylene tubes via a lumbar puncture (LP) in left-lateral supine position at the lower lumbar vertebrae using an atraumatic 20-gauge needle (Sprotte, Pajunk, 90–120 mm length) after local anesthesia with 2% lidocaine (42, 47). In case lumbar puncture in supine position is unsuccessful after two attempts, a final attempt in prone position will be performed. Within 60 min of obtaining the CSF, erythrocytes and leukocytes counts will be determined. Within 2 h after collection, the CSF is centrifuged, aliquoted in polypropylene tubes, and stored at −80 °C until further use.

We will draw 10 mL serum and 30 mL plasma EDTA samples via venepuncture from all participants at the baseline and follow-up visits. Blood will be immediately transported for sample processing, aliquoting, and storage at −80 °C until further use. At baseline, DNA for genotyping will be isolated from whole blood collected in a separate EDTA tube. The genotyping is targeted to specifically determine the variants of the *APOE* gene (48).

One week after each visit the participant will be contacted to inform if any visit-related complaints have developed.

Analysis of the total protein concentration and albumin concentration will be carried out with an Atellica NEPH 630 analyser in CSF and serum. Analysis of the two longitudinal samples, from the baseline and follow-up visit, will be performed for the following parameters within the same batch to minimize batch effects: Aβ40, Aβ42, t-tau, and p-tau (phosphorylated at Thr181) will be analysed in CSF using a Lumipulse Chemiluminescent immunoassay (Fujirebio, Ghent, Belgium). Aβ38 and Aβ43 in CSF will be analysed as previously described (17). Potential BBB-dysfunction biomarkers are evaluated based on: (1) BBB-specificity (e.g., based on single cell mRNA expression, immunohistochemistry of brain tissue), (2) involvement in BBB functionality (e.g., tight-junction, cell–cell communication, inflammation), (3) measurability in CSF and/or plasma, and (4) availability of suitable immunoassays (21). Information concerning potential biomarker candidates was obtained from curated datasets and literature. An overview of evaluated proteins is provided in the Supplementary Table S5. Currently, we envision to measure longitudinally the CSF concentrations of TIE2, MCAM, and VCAM1, as based on our preliminary findings (21). Analysis of fluid BBB-dysfunction biomarkers will be performed with immunoassays. Assays will be validated as previously described (49).

#### Statistical methods

For our primary aim, we will compare the longitudinal changes in CSF Aβ-peptide levels between patients with CAA and healthy, age- and sex-matched control participants. Data are expected to follow a normal distribution, and parametric tests will be used to assess differences between groups. If the data are not normally distributed, a natural logarithmic transformation will be applied to obtain a normal distribution. If this approach does not suffice, we will describe the distribution of the peptide changes using the median and interquartile range and subsequently apply appropriate non-parametric tests.

We will assess longitudinal differences between the study groups using linear mixed-effects models. Interval differences, age and *APOE* genotype will be included as covariates to account for their potential confounding effects.

##### Secondary study parameters

The MRI-based BBB integrity measurements are continuous variables, expressed as *K_i_*, *V_i_*, and *V_p_*, and will be calculated across the whole brain as well as in separate cortical (temporal and occipital lobe). In case of non-normal data distributions, log-transformations will be performed where necessary. We assume a linear relationship between *K_i_* and *V_i_* and the Aβ-peptides levels, fluid biomarkers of BBB-dysfunction (both novel and the Qalb), and the MRI markers of SVD. These associations will be assessed using multivariable linear regression models, adjusted for age and APOE genotype. To account for multiple comparisons, *p*-values will be adjusted using the Benjamini–Hochberg false discovery rate (FDR) procedure, with an FDR threshold of 0.05.

Biochemical assays to quantify fluid BBB-dysfunction biomarkers will first be evaluated according to technical characteristics (49). Thereafter, the groups will be compared using univariate parametric or non-parametric tests, as appropriate. Correlations between the Aβ peptides, emerging BBB-biomarkers, clinical symptoms (as addressed in the questionnaire), cognitive function, and MRI-vascular related results will be assessed using linear regression analysis with age and *APOE* genotype as covariates. Longitudinal changes in CSF biomarkers will be analysed using linear mixed-effects models, which will also allow inclusion of participants with incomplete follow-up measurements under the assumption that data are missing at random. This assumption is justified since failure of a lumbar puncture or MRI acquisition is independent of the study group. Linear mixed-effects models use all available observations and avoid excluding participants with partially missing longitudinal data. The extent and patterns of missing data will be reported descriptively. As a sensitivity analysis, characteristics of participants with complete and incomplete follow-up data will be compared to assess the potential impact of attrition on study findings.

#### Anticipated setbacks and coping strategies

Several potential setbacks have been evaluated prior to the start and during interim evaluations of the study. In this paragraph we address our foreseen setbacks and coping strategies.

A lower-than-anticipated recruitment rate of CAA participants is not expected since within the outpatient neurology clinic of the Radboudumc annually approximately 50 to 60 newly diagnosed patients with probable CAA are seen. In case the recruitment rate of control participants is lower than expected we can increase the approach rate of registered individuals in ‘hersenonderzoek.nl/’ which contains more than 40,000 citizens interest in participating in brain research.

Incomplete study procedures of participants, either due to an unsuccessful lumbar puncture, incomplete MRI acquisition, and a MOCA score below 27 in control participants, will result in researcher-initiated withdrawal of the participant. A replacement participant will be recruited.

## Discussion

This longitudinal study will provide novel insights into CAA progression by evaluating changes in CSF Aβ-peptide concentrations and their association with of BBB dysfunction, assessed both with imaging and in CSF. This interdisciplinary study evaluates the CSF profile of Aβ peptides, novel fluid BBB-dysfunction biomarkers and complementary MRI-based leakage assessment. With this approach, we aim to further elucidate the relation between temporal changes of clinical disease progression, underlying biochemical pathophysiology, and cross-sectionally image-based BBB-impairment in CAA and in controls. We hypothesize that the CSF Aβ38, Aβ40, Aβ42, and Aβ43 concentrations will notably decline over 2 years in CAA compared to controls (17). Moreover, we expect no major longitudinal differences of these Aβ peptides in controls, consistent with previous finding (17, 50).

The assessment of BBB dysfunction using two orthologous methods, DCE-MRI and fluid biomarkers, will provide additional insights into their utility and biological relevance in a clinical setting. To date, the first two explorative DCE-MRI studies have reported conflicting findings. Whereas increased leptomeningeal vessel leakage was described in CAA in one study (33), the other has reported decreased BBB leakage (34). The present study not only aims to extend on these previous studies but also aims to complement these measurements with fluid BBB biomarkers. We expect to improve our knowledge of the BBB dysfunction by a critical evaluation of currently used biomarkers such as the albumin ratio and our proposed novel fluid BBB biomarkers, against DCE-MRI permeability in both CAA and healthy controls.

The inclusion of age- and sex-matched control participants without CAA introduces an unavoidable uncertainty: these participants could have asymptomatic amyloid pathologies either in the vasculature and/or as plaques in the parenchyma. Since in cognitive normal elderly the prevalence of CAA pathology is low (5–7%), and a cognitive test is included in our inclusion criteria, we expect to include at most two people with asymptomatic CAA in the control group (15). This is unlikely to significantly influence the conclusions of our study. Another unavoidable bias is the drop-out of participants over time. We assume that the loss-to-follow-up is low but predominantly occurring with more severely impaired CAA participants, and this may skew our follow-up analyses towards the less severely affected people. The stringent decision to exclude cognitively impaired controls is justified since it will reduce the chance of including participants with underlying CAA (or other neurological diseases) in the control group as cognitive decline can be a presenting symptom of CAA, and we aim to avoid this potential information bias.

At last, we were unable to perform a formal power calculation since longitudinal data of CSF Aβ peptides in sporadic CAA is unavailable. This highlights the importance of this explorative study, where we expect to generate such longitudinal data. In a previously published study, it was shown that in a hereditary CAA variant, i.e., Dutch type CAA which is fully penetrant, the CSF levels of Aβ40 and Aβ42 were already decreased in pre-symptomatic carriers as compared controls, implying that during disease development CSF levels of Aβ may change (17). This supports the goals of our observational study.

The MODEM-LAMBDA-C study will provide multidisciplinary insights on longitudinal fluid biomarkers for CAA and BBB dysfunction. These advancements will contribute to our understanding of this disease for improved diagnostics and treatment decision-making.
