# Supplementary material for: Longitudinal amyloid-beta measurements and blood–brain barrier dysfunction in cerebral amyloid angiopathy as part of the Mechanisms Of DEMentia project: protocol of the MODEM-LAMBDA-C study
Source: Front Neurol. 2026 Oct 28;17:1949765. doi: 10.3389/fneur.2026.1949765 (PMC13753768; doi:10.3389/fneur.2026.1949765)
Supplement: Supplementary file 1 [file Supplementary_file_1.docx]

## Supplementary information

Supplementary table S1. Inclusion and exclusion criteria per research group

| Participants with CAA | |
| --- | --- |
| Inclusion criteria | |
| 1. | Age > 50 years |
| 2. | Written informed consent |
| 3. | Subjects are mentally competent to take a decision on participation |
| 4. | Fulfilment of the Boston criteria 2.0 for probable CAA (32) |
| 5. | Most recent ICH was at least 6 months ago (if ICH is applicable) |
| 6. | *In case of prior participation in the BIONIC/CAFÉ study:* the lumbar puncture at baseline visit successfully acquired CSF |
|  | |
| Exclusion criteria | |
| 1. | Presence of blood coagulopathy |
| 2. | History of major psychiatric disorder |
| 3. | Pregnancy at time of study participation |
| 4. | Allergy to local anaesthetic agents |
| 5. | Contra-indication for lumbar puncture: medical history of compression of spinal cord, spinal surgery, skin infection, developmental abnormalities in lower spine |
| 6. | Subjects who are currently participating in another study or have participated in a clinical study within the previous 30 days, based on their own report |
| 7. | Subjects with a history of or current drug or alcohol (>14 consumptions per week) abuse |
| 8. | Subjects who are part of the study staff personnel or family members of the study staff |
| 9. | Proven mutation carrier for D-CAA |
| 10. | Contra-indications for MRI* |
|  | |
| Control participants | |
| Inclusion criteria | |
| 1. | Age > 50 years |
| 2. | Written informed consent |
| 3. | Subjects are mentally competent to take a decision on participation |
| 4. | A MoCA score ≥27 (34) |
| 5. | *In case of prior participation in the BIONIC/CAFÉ study:* the lumbar puncture at baseline visit successfully acquired CSF |
|  | |
| Exclusion Criteria | |
| 1. | Presence of blood coagulopathy |
| 2. | History of major psychiatric disorder |
| 3. | Pregnancy at time of study participation |
| 4. | Allergy to local anaesthetic agents |
| 5. | Contra-indication for lumbar puncture: medical history of compression of spinal cord, spinal surgery, skin infection, developmental abnormalities in lower spine |
| 6. | Subjects who are currently participating in another study or have participated in a clinical study within the previous 30 days, based on their own report |
| 7. | Subjects with a history of or current drug or alcohol (>14 consumptions per week) abuse |
| 8. | Subjects who are part of the study staff personnel or family members of the study staff |
| 9. | Proven mutation carrier for the D-CAA |
| 10. | Contra-indications for MRI* |
| 11. | Self-reported(subjective) cognitive decline |
| 12 | history of major neurological (stroke, neurodegenerative disease, brain tumours, brain infection or inflammation) |

**MRI-contra indications:

Claustrophobia

Pacemaker or implantable cardioverter-defibrillators (ICDs)

Neurostimulators

Intraorbital or intraocular metallic foreign bodies/fragments

Cochlear implant

Ferromagnetic implants

Hydrocephalus shunt

Intrauterine device (IUD)

Orthodontic wire place behind the teeth prior to 1995

Permanent makeup

Tattoos above shoulder level

Severe physical limitation or inability to undergo MRI scanning

Body weight exceeding 120 Kg

Inability to remain lying still for 60 minutes

Impaired renal function (eGFR < 30 mL/min)

Supplementary table S2. Inclusion and exclusion criteria per research group of the previously conducted CAFÉ/BIONIC studies

| Participants with CAA | |
| --- | --- |
| Inclusion criteria | |
| 1. | Age > 55 years |
| 2. | Written informed consent |
| 3. | Subjects are mentally competent to take a decision on participation |
| 4. | Fulfilment of the Boston criteria 1.5 for probable CAA (32) |
| 5. | Most recent ICH was at least 3 months ago (if ICH is applicable) |
|  | |
| Exclusion criteria | |
| 1. | Presence of blood coagulopathy |
| 2. | History of major psychiatric disorder |
| 3. | Pregnancy at time of study participation |
| 4. | Allergy to local anaesthetic agents |
| 5. | Contra-indication for lumbar puncture: medical history of compression of spinal cord, spinal surgery, skin infection, developmental abnormalities in lower spine |
| 6. | Subjects who are currently participating in another study or have participated in a clinical study within the previous 30 days, based on their own report |
| 7. | Subjects with a recent history of or current drug or alcohol (>14 consumptions per week) abuse |
| 8 | Subjects with a recent history of or current drug abuse |
| 9. | Subjects who are part of the study staff personnel or family members of the study staff |
| 10. | Proven mutation carrier for D-CAA |
| 11. | Contra-indications for MRI* |
| 12. | Recent (<3 months) symptomatic stroke |
| 13. | Intracranial clips |
|  | |
| Control participants | |
| Inclusion criteria | |
| 1. | Age > 55 years |
| 2. | Written informed consent |
| 3. | Subjects are mentally competent to take a decision on participation |
| 4. | A MoCA score >28 |
|  | |
| Exclusion Criteria | |
| 1. | Presence of blood coagulopathy |
| 2. | History of major psychiatric disorder |
| 3. | Pregnancy at time of study participation |
| 4. | Allergy to local anaesthetic agents |
| 5. | Contra-indication for lumbar puncture: medical history of compression of spinal cord, spinal surgery, skin infection, developmental abnormalities in lower spine |
| 6. | Subjects who are currently participating in another study or have participated in a clinical study within the previous 30 days, based on their own report |
| 7. | Subjects with a recent history of or current drug or alcohol (>14 consumptions per week) abuse |
| 8. | Subjects with a recent history of or current drug abuse |
| 9. | Subjects who are part of the study staff personnel or family members of the study staff |
| 10. | Contra-indications for MRI* |
| 11. | Self-reported(subjective) cognitive decline |
| 12. | history of major neurological (stroke, neurodegenerative disease, brain tumours, brain infection or inflammation) |
| 13. | Intracranial clips |

**MRI-contra indications:

Claustrophobia

Pacemaker or implantable cardioverter-defibrillators (ICDs)

Neurostimulators

Intraorbital or intraocular metallic foreign bodies/fragments

Cochlear implant

Ferromagnetic implants

Hydrocephalus shunt

Intrauterine device (IUD)

Orthodontic wire place behind the teeth prior to 1995

Permanent makeup

Tattoos above shoulder level

Severe physical limitation or inability to undergo MRI scanning

Body weight exceeding 120 Kg

Inability to remain lying still for 60 minutes

Impaired renal function (eGFR < 30 mL/min)

*Supplementary table S3: interim recruitment overview of the MODEM-LAMBDA-C study, as of mid-October 2026.*

|  | Control | CAA |
| --- | --- | --- |
| n contacted for recruitment | 85 | 45 |
|  |  |  |
| n finished study * | 12 | 14 |
| Interval period (median years, [IQR])* | 4.24 [4.01 – 4.45] | 3.50 [2.74 – 4.10] |
| n baseline visit successful** | 12 | 3 |
|  |  |  |
| Prematurely stopped | 9 | 3 |
| Failed LP | 2 | 2 |
| MOCA excluded | 7 | n.a. |
| Incomplete MRI | 0 | 1 |

*The numbers refer to former BIONIC/CAFÉ participants who have also participated in the MODEM-LAMBDA-C study. No newly recruited MODEM-participants have had a follow-up visit yet.
** The numbers refer to newly recruited MODEM-LAMBDA-C participants

Supplementary table S4: acquisition details MRI sequences

|  | QSM | T1w | FLAIR | DWI | DCE | | |
| --- | --- | --- | --- | --- | --- | --- | --- |
| Sequence | 3D SPGR | 3D MP2RAGE | SPACE | Resolve | Pre-bolus slow T1w SR SPGR | Fast T1w SR SPGR | Post-bolus slow T1w SR SPGR |
| Orientation | Sag | Sag | Sag | Tra | Sag | Sag | Sag |
| Voxel size (mm) | 1.0×1.0×1.0 | 1.0×1.0×1.0 | 0.5x0.5x1.0 | 0.6x0.6x5.0 | 1.0x1.0x2.0 | 1.3x1.3x3.0 | 1.0x1.0x2.0 |
| TR (ms) | 40 | 6000 | 5000 | 3040 | 396 | 313 | 296 |
| TE (ms) | 3.48, 8.67, 13.86, 19.05, 24.24, 29.43, 34.62 | 1.71 | 394.0 | 61.0, 108.0 | 1.56 | 1.45 | 1.56 |
| TI (ms) | N.A. | 900, 2200 | 1600 | N.A. | 120 | 120 | 120 |
| FLIP angle (°) | 15 | 6, 5 | T2 var | 180 | 20 | 20 | 20 |
| Number of slices | 176 | 176 | 176 | 28 | 60 | 10 | 60 |
| FOV | 256 | 256 | 250 | 230 | 256 | 320 | 256 |
| Acc | 6 | 3 | 2 | 4 | 3 | 2 | 3 |
| NM | 1 | 1 | 1 | 1 | 5 | 32 | 70 |
| TA (mm:ss) | 5:08 | 7:50 | 4:47 | 1:33 | 1:47 | 2:00 | 24:52 |

Abbreviations: FLAIR = Fluid-attenuated inversion recovery; DWI = diffusion-weighted imaging; DCE = Dynamic contrast-enchanced; MPRAGE= Magnetization-prepared rapid acquisition with gradient echo; MP2RAGE = Magnetization-prepared 2 rapid acquisition with gradient echo; SR = saturation recovery; SPGR = spoiled gradient recalled echo; Sag = sagittal; Tra = transversal; TR = retention time; TE = Echo Time; TI = inversion time; N.A. = not applicable; FOV = field of view; Acc = parallel imaging acceleration factor; NM= number of measurements; TA = acquisition time

Supplementary table S5: Selected BBB-biomarkers to be studied within MODEM-LAMBDA.

| **research interest** | **Gene name** | **BBB** | **BCSF-B** | **Neurons** | **immunoassay** |
| --- | --- | --- | --- | --- | --- |
| h | ANGPT2 | +++ | - | - | + |
| h | CAV1 | ++ | - | + | + |
| h | CD34 | +++ | - | - | ? |
| h | CDH5 | +++ | - | - | + |
| h | ESAM | +++ | - | - | + |
| h | ICAM1 | +++ | - | - | + |
| h | MCAM | +++ | - | - | + |
| h | MFSD2A | + | - | - | - |
| h | MMRN2 | + | - | - | - |
| h | PECAM | +++ | - | - | + |
| h | PODXL1 | ++ | - | - | ? |
| h | TEK | ++ | - | - | + |
| h | VCAM | + | - | - | + |
| h | VWF | +++ | - | - | + |
| m | CD13 | +++ | ? | ? | ? |
| m | EDN1 | + | ? | - | ? |
| m | F11R | + | + | + | + |
| m | ITIH4 | + | ? | +++ | ? |
| m | JUP | + | ++ | - | ? |
| m | OCLN | +++ | +++ | + | + |
| m | PDGFA | ++ | + | + | + |
| m | PDGFB | +++ | - | + | + |
| m | SELE | + | - | - | + |
| m | SMOC2 | ++ | + | - | ? |
| m | SNAI2 | ++ | ? | + | ? |
| m | TAGLN2 | +++ | ? | - | + |
| m | TJP1 | + | + | + | + |
| m | TJP2 | + | ++ | + | + |
| m | VEGFC | +++ | ? | ++ | ? |
| l | ADAMTS6 | +++ | +++ | +++ | ? |
| l | ANGPT1 | +++ | +++ | - | ? |
| l | BSG | +++ | +++ | - | + |
| l | CLDN5 | +++ | ++ | - | + |
| l | DAG1 | +++ | ++ | + | ? |
| l | ENG | +++ | - | ? | + |
| l | HP | ++ | - | - | + |
| l | PGP | ++ | + | ++ | ? |
| l | PLVAP | - | + | - | + |

Legend: BBB: expression expected in blood-brain barrier; BCSFB, expression expected in the blood-CSF barrier; neurons: expected expression by neurons; immunoassay: availability of immunoassays for protein quantification. h, high interest; m, medium interest; l, low interest; - , no evidence found; + some expression; ++, moderate expression; +++, high expression; ?, information unknown.
