# Supplementary material for: Longitudinal amyloid-beta measurements and blood–brain barrier dysfunction in cerebral amyloid angiopathy as part of the Mechanisms Of DEMentia project: protocol of the MODEM-LAMBDA-C study
Source: Front Neurol. 2026 Oct 28;17:1949765. doi: 10.3389/fneur.2026.1949765 (PMC13753768; doi:10.3389/fneur.2026.1949765)
Supplement: Supplementary file 2 [file Supplementary_file_2.docx]

Supplementary 1 questionnaire clinical symptoms MODEM-LAMBDA-C study

At the baseline visit of the MODEM-LAMBDA-C study, the following questions will be asked to each participant:

1. Symptoms:

Have you ever experienced a haemorrhage: yes/no

If yes, how many haemorrhages?

Date of the last haemorrhage:

Do you have cognitive complains yes no,

If yes, has dementia been diagnosed? Yes/no

If yes, when was this diagnosed?

2. Associated conditions

Have you ever had the diagnosis of a:

- A significant head trauma:	yes/no/unknown
- Episodes of migraine:	yes/no/unknown
- Sleeping disorder	:	yes/no/unknown
- An intracranial neurosurgery:	yes/no/unknown
- Intracranial radiotherapy:	yes/no/unknown

3. Family-anamnesis

Has anyone in your family been diagnosed with any of the following conditions? If so, please indicate who and at what age.

Dementia: yes/no/unknown

Stroke: yes/no/unknown

4. Vascular risk factors

Atrial fibrillation:	yes/no/unknown

Hypertension:	yes/no/unknown

Hypercholesterolemia:	yes/no/unknown

Type 2 diabetes mellitus:	yes/no/unknown

Myocardial infarction:	yes/no/unknown

Height (m):

Weight (Kg):

Smoking:	no never/ no, but is former smoker/current

Number of packyears:

If former smoker, since:

Alcohol consumption: no never/ no, but is former user/current

Number of units per week:

If former consumer, since:

Drug use: no never/ no, but is former user/current

If former user or current: which drugs

Number of units per week:

If former user, since:

5. Overview of current medication

At the follow-up visit, the following questions will be asked:

1. Symptoms

Since the last visit, have you had a haemorrhage?	Yes/no/unknown

If so, how many?

Date last haemorrhage:

Since the last visit, have there been cognitive complains?	Yes/no/unknown

If yes, has dementia been diagnosed?	Yes/no

If yes, when was this diagnosed?

Have you experienced any transient neurological deficits since your last visit?

Yes/no
